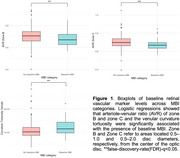# Retinal microvasculature alterations are associated with mild behavioral impairment in a memory clinic population

**DOI:** 10.1002/alz70861_108343

**Published:** 2025-12-23

**Authors:** Yingqi Liao, Ming Ann Sim, Xin Xu, Yih Chung Tham, Christopher Chen

**Affiliations:** ^1^ Memory, Ageing, and Cognition Centre (MACC), Department of Pharmacology, Yong Loo Lin School of Medicine, National University of Singapore, Singapore Singapore; ^2^ Department of Anesthesia, National University Health System and National University of Singapore, Yong Loo Lin School of Medicine, Singapore Singapore; ^3^ School of Public Health and the Second Affiliated Hospital of School of Medicine, Zhejiang University, Hangzhou, Zhejiang China; ^4^ Centre for Innovation and Precision Eye Health, and Department of Ophthalmology, Yong Loo Lin School of Medicine, National University of Singapore, Singapore Singapore; ^5^ Ocular Epidemiology Research Group, Singapore Eye Research Institute, Singapore National Eye Centre, Singapore Singapore; ^6^ Ophthalmology and Visual Science Academic Clinical Program, Duke‐NUS Medical School, Singapore Singapore; ^7^ Department of Psychological Medicine, Yong Loo Lin School of Medicine, National University of Singapore, Singapore, Singapore Singapore

## Abstract

**Background:**

Mild behavioral impairment (MBI) is characterized by the late‐life emergence of neuropsychiatric symptoms (NPS) as a prodromal marker of dementia. While its biological mechanisms remain unclear, emerging evidence suggests an association between cerebrovascular pathology and MBI. Considering the close connection between retinal and cerebral microvasculature, this study aimed to investigate retinal microvascular changes in MBI among dementia‐free older adults.

**Method:**

The study included 306 participants ‐ mean age:71.3±7.8 years old, 53%(*n* =161) female, 68%(*n* =207) Cognitively impaired‐No dementia (CIND) ‐ who underwent neuropsychological assessments and fundus photography examination at baseline and during annual follow‐up over a maximum period of five years. Baseline MBI was ascertained using the Neuropsychiatric Inventory (NPI) at baseline and Y1. For individuals without baseline MBI, incident MBI was defined as the presence of NPS (NPI≥1) in any two consecutive years over follow‐up. Retinal vascular features (caliber, tortuosity and fractal dimension) were quantitatively measured using semi‐automated software. Logistic regression was employed to examine the associations between retinal biomarker z‐scores and baseline MBI. Competing risk regression was conducted to examine links between retinal biomarkers with incident MBI longitudinally, with incident dementia as the competing risk event. All models were adjusted for age, sex, education and diagnostic groups (i.e. no cognitive impairment *vs* CIND) at baseline. The significance level of tests was set at *p* <0.05, with false discovery rate correction (q<0.05) applied for multiple testing.

**Result:**

Baseline MBI (*n* =51) was significantly associated with reduced arterio‐venule‐ratio (AVR) of zone B (per SD increase, OR=0.64; 95% CI, 0.45‐0.89) and zone C (per SD increase, OR=0.59; 95% CI, 0.41‐0.82) and increased venular curvature tortuosity (per SD increase, OR =1.48; 95%CI, 1.12‐1.98) (Figure 1). Furthermore, reduced zone C‐AVR was independently associated with a higher likelihood of incident MBI (*n* =50) (per SD increase, sHR=0.75; 95%CI, 0.59‐0.95), after accounting for incident dementia (*n* =19). Notably, this relationship between zone C‐AVR and incident MBI was independent of baseline diagnostic group (interaction *p* >0.05).

**Conclusion:**

Our findings demonstrated retinal microvascular alterations associated with baseline and incident MBI, offering important insights into the pathophysiology of early behavioral disturbances preceding dementia.